# Temporal dynamics for areal unit-based co-occurrence COVID-19 trajectories

**DOI:** 10.3934/publichealth.2022049

**Published:** 2022-10-14

**Authors:** Gabriel Owusu, Han Yu, Hong Huang

**Affiliations:** 1 Department of Applied Statistics and Research Methods, University of Northern Colorado, Greeley, CO 80639, USA; 2 School of Information, University of South Florida, Tampa, FL, 33620, USA

**Keywords:** latent log-linear Poisson process, temporal dynamics, COVID-19, hierarchical models, co-occurrence

## Abstract

The dynamic mechanism of the COVID-19 pandemic has been studied for disease prevention and health protection through areal unit-based log-linear Poisson processes to understand the outbreak of the virus with confirmed daily empirical cases. The predictor of the evolution is structured as a function of a short-term dependence and a long-term trend to identify the pattern of exponential growth in the main epicenters of the virus. The study provides insight into the possible pandemic path of each areal unit and a guide to drive policymaking on preventive measures that can be applied or relaxed to mitigate the spread of the virus. It is significant that knowing the trend of the virus is very helpful for institutions and organizations in terms of instituting resources and measures to help provide a safe working environment and support for all workers/staff/students.

## 1. Introduction

COVID-19 has spread throughout the world. The pandemic has had a significant impact on human life and the economy. In the course of history, as mankind became more civilized, constructing routes to connect nations and cities and forging inter-city and international trades, more pandemics began to spread quickly when there was an outbreak. Humans are not the only living organism that experiences pandemics, as other living organisms such as plants and animals also at some point have experienced some form of a pandemic. Some of the pandemics in the last century include the Spanish flu, Asian flu, AIDS pandemic, H1N1 flu pandemic, West Africa Ebola epidemic, the ZIKA virus epidemic and now the novel coronavirus (COVID-19). Coronaviruses were initially found to be caused by an infectious bronchitis virus in the 1930s resulting from an acute respiratory infection of domesticated chickens [12].

In the 1960s, human coronaviruses were found [16]. In the UK and the United States of America, they were isolated by two processes [24]. A new common cold virus B814 was isolated from a boy by Kendall, Malcom Byone and David Tyrrell from the common cold unit of the English Medicine Board in 1960 [24]. The virus could not be cultivated using standard techniques, i.e., with rhinoviruses, adenoviruses and other known frequent cold viruses that are successfully grown. Bypassing it through the organ culture of the human embryonic trachea in 1965, Tyrrell and Byone cultivated the new virus successfully [35]. A new method of cultivation was introduced [20]. The isolated virus inoculated in volunteers caused a cold and was inactivated in a lipid envelope by ether.

More than 536 million confirmed cases worldwide, including almost 6.3 million deaths, have been recorded since its discovery in December 2019 to June 2022 [36] COVID-19's apparent ability to easily spread and cause serious disease in older adults and patients with existing medical conditions are worrying [19]. Coronaviruses are famous for modifying and recombining [29]. Since the first report, the SARS-CoV-2 genomic sequence has changed. Some scientists believe that there are two main circulating strains of the virus, i.e., the deadly strain “L” and less virulent strain “S” [34].

No specific antiviral therapies or COVID-19 vaccines were available in the beginning. Therapies have focused mainly on symptomatic and respiratory support according to the protocols issued in the various countries, which were following the World Health Organization protocols issued by the health authorities [19]. In the 21st century, following a major respiratory syndrome in 2003, COVID-19 has become the third novel coronavirus to cause a widespread epidemic [23].

COVID-19 has a high level of transmission, not only due to the social and economic risk factors [4],[6],[7],[9],[17], but also through sewage, watercourses and other environmental compartments [31]. Recent research found that countries with good governance and high public health spending implemented the vaccination campaign more quickly and were more successful in controlling the spread of infection and preventing the collapse of the national health system [3]. At the city level, similar findings have shown that the more developed cities and areas showed lower risk of death from COVID-19 [37]. COVID-19 appears to have created conditions favorable to policy changes, driving improvements in crisis preparedness [8]. Recent research also showed that socioeconomic factors, ethnicity, education and inequality impact county-level COVID-19 mortality [21]. Lockdown policy measures hinder mobility [2], and the frequency of patients and severe symptoms requiring hospitalization rise with age [32]. Differences in Asian and European epidemiology may impact disease spread and mortality [39].

To assess the effects of the preventive measures already taken in different areas and eventually prevent the COVID-19 pandemic from effectively spreading, it is useful to initiate an areal unit-based study of the dynamics of COVID-19 to explore and understand the dynamic process of virus outbreak through areal unit-based temporal modeling that is amenable for developing further theoretical analysis in hierarchical dynamic spatiotemporal modeling and inference; this is because it would allow for examining multiple areal units and considering multivariate interaction. In contrast to the previous studies that used the data of earlier periods of the spread, i.e., before we have reached equilibrium and when most governments, institutions and organizations were not ready to take intervention measures when vaccines were not popular, we chose to use the daily COVID-19 case data most recently available until June 2022 with a wider time span; thus, the data can include the up-to-date information regarding preventive intervention measures such as social distancing and vaccination and their effectiveness. We also trained the areal unit-based models for different countries under unobserved heterogeneous environments in our study to obtain more robust scientific insight into the nonlinear dynamics of the outbreak of the COVID-19 pandemic, which has resulted in enormous personal and societal losses. The dynamics learned from the richer empirical cases available until June 2022 can be applied to illustrate close or exact values for new cases in areal units, assess potential intervention assistance in reducing virus spread and drive policy decisions regarding virus spread mitigation.

The rest of the paper is organized as follows. Section 2 introduces the COVID-19 data collection method and variables in the study. The proposed models and methods for the study are presented in Section 3. Our findings are summarized in Section 4, followed by the discussion in Section 5. The paper ends with conclusions in Section 6.

## 2. Data collection and variables

The data source is the COVID-19 Data Repository of the Center for Systems Science and Engineering at Johns Hopkins University, from which we extracted the total confirmed cases, confirmed deaths and testing information from January 31, 2019 to June 30, 2022. Table 1 provides a description of the extracted datasets for the study with their columns.

**Table 1.** Datasets.

**Dataset**	**Descriptions**	**Columns**
time_series_covid19_confirmed_global.csv	Time-series data of confirmed cases	Province/state, Country/Region Latitude, Longitude, Date.
time_series_covid19_deaths_global.csv	Time-series data of death cases	Province/state, Country/Region Latitude, Longitude, Date.
time_series_covid19_recovered_global.csv	Time-series data of recovered cases	Province/state, Country/Region Latitude, Longitude, Date.

Table 1 shows variable descriptions of the datasets that were used for the study. The first dataset was the total confirmed cases of COVID-19, where the counts included probable cases, where reported. The second dataset was the total death cases reported, and the last dataset was counts of recovered cases. All datasets start from the time that the first case was recorded and end on June 30, 2022. Each dataset contained the covariate information of the province/state, country/region latitude and longitude where the cases were recorded. Province/state is the province or state of the observation, country/region is the country or region of observation, latitude is the latitude of the observed country or region, longitude is the longitude of the observed country or region and date gives the dates of confirmed cases/deaths/recovered cases that were reported till June 30, 2022.

## 3. Models and data analysis procedure

Many mathematical and statistical models and methods have been applied to study the dynamics of COVID-19. Specifically, regression analysis was conducted for the effects of social and economic risk factors [13],[22],[27],[33],[40], time-series models were utilized to deal with temporal dependency throughout the pandemic [25],[5] and spatial models have been employed for understanding the diffusion of COVID-19 spread [10]. Other recent studies attempted to build efficient methods that incorporate both time and space effects in order to model COVID-19's dynamics [11],[28],[30],[38].

Agosto and Giudici ([1]) employed a Poisson autoregressive (PAR) model to understand COVID-19 contagion dynamics. The earlier cases of COVID-19 from China, Iran, South Korea and Italy were used to train the three different models: classical exponential model, PAR model and PAR model with covariates. The root mean square error and mean percentage error were used for model selection from the three considered models. The PAR model outperforms the other two models, and this is consistent with our model for this study. Kharroubi ([26]) worked on modeling and predicting the spread of COVID-19 in Lebanon from a Bayesian alternative. Two different models were proposed and implemented by using Bayesian Markov chain Monte Carlo simulation methods: a PAR model as a function of a short-term dependence only, and a PAR model as a function of both a short-term dependence and a long-term dependence. The two models were compared in terms of their predictive ability by using the root mean square error and deviance information criterion (DIC). The PAR models that allow the capture of both short- and long-term memory effects performed better for all criteria.

**• Log-Linear Poisson Model**

The reported count of new cases *Y_t_* at day *t* in the areal unit *s* was assumed to follow a conditional Poisson distribution:

$$Y_{st}|{ℱ}_{s,t-1}^{Y,\lambda }∼Poisson\left(\lambda_{st}\right),$$

where $F_{s,t-1}^{Y,\lambda }$ denotes the σ-field generated by $\left\{Y_{s0},...,Y_{s,t-1},\lambda_{s0}\right\}$, i.e., $F_{st}^{Y,\lambda }=\sigma \left(Y_{sr},r\leq t,\lambda_{s0}\right)$, and {*λ_st_*} is a Poisson intensity process with the dependence structure

$$log\left(\lambda_{st}\right)=k+\alpha log\left(1+Y_{s,t-1}\right)+\beta log\left(\lambda_{s,t-1}\right),$$ (1)

where *k* ∈ *R* is the intercept term, *α* ∈ *R* and *β* ∈ *R* express the dependence of the expected number of new infections, *λ_st_*, on the past observed counts *y_st_*_−1_ of new infections and the past expected number of infections *λ_s,t_*_−1_ in the areal unit *s*, respectively. In other words, the short-term dependence is captured by *α*, while the long-term trend on all past values of the observed process is represented by *β*. Model (1) is expected to be more parsimonious than the model which includes higher lags of *log*(*Y_st_* + 1), but without the feedback mechanism introduced by *λ_st_*. The inclusion of the *β* component is analogous to moving from an ARCH (autoregressive conditionally heteroscedastic, Engle 1982) to a GARCH (generalized autoregressive conditionally heteroscedastic, Engle and Bollerslev 1986) model using Gaussian processes, and it allows us to capture long-term memory effects.

Additionally, *log*(1 + *Y_st_*_−1_), instead of *log*(*Y_s,t_*_−1_), is included to avoid the ill-defined number on the days when there are no reported cases. The log-linear autoregression predictor (1) can accommodate both positive and negative association, as well as include time-dependent covariates in a straightforward manner.

If *Y_st_ is* formulated as the number of events *N_st_*(*λ_st_*) of a Poisson process *N_st_*(*·*) of unit intensity in the time interval [0, *λ_t_*] for each time point t in the areal unit *s*; then, *Y_st_* can be explicitly considered as samples from a sequence of independent Poisson processes of unit intensity {*N_st_*(*·*), t=1, 2, ...} given $v_{st}\equiv log\lambda_{st}$. The model (1) is therefore represented as the following hierarchical log-linear autoregressive model (*Y_st_*, *ν_st_*):

$$Y_{st}=N_{st}\left(\lambda_{st}\right),v_{st}=d+av_{s,t-1}+blog\left(Y_{s,t-1}+1\right),t\geq 1,$$ (2)

where both *ν_s_*_0_ and *Y_s_*_0_ are fixed in the areal unit *s*. The first part of (2) is the observation model, and the second part of (2) is the latent process model for the underlying dynamic mechanisms. The parameters *d*, *a*, *b* belong to *R*, but restrictions on the parameter space can be imposed so that a central limit theory for {(*Y_st_*, *ν_st_*)} can be developed. The choice of the log function for the lagged values of the response *Y_s,t_*_−1_ is based on the following consideration.

Consider a model like (2), but with *Y_s,t_*_−1_ included instead of *log*(*Y_s,t_*_−1_ + 1):

$$Y_{st}|F_{s,t-1}^{Y,v}\sim Poisson\left(\lambda_{st}\right),v_{st}=d+av_{s,t-1}+bY_{s,t-1};$$

then,

$$\lambda_{st}=exp\left(d\right)\lambda_{s,t-1}^{a}exp\left(bY_{s,t-1}\right).$$

Therefore, the stability of the above system is guaranteed only when *b* < 0. Otherwise, the process *λ_st_* increases exponentially fast. Hence, only a negative correlation can be introduced by such a model. However, (2) allows for a positive (negative) correlation by allowing the parameter *b* to take positive (negative) values.

We will work with the latent canonical link process {*ν_st_*}. Note that the latent areal unit-based log-intensity process {*ν_st_*} of (2) is expressed as

$$v_{st}=d\frac{1-a^{t}}{1-a}+a^{t}v_{s0}+b\overset{t-1}{\underset{i=0}{\sum }}a^{i}log\left(1+Y_{s,t-i-1}\right)$$ (3)

after repeated substitution. Therefore, the hidden process model *ν_st_* is determined by past functions of lagged responses. Both the data process {*Y_t_*} and the latent process {*λ_t_*} given areal unit *s* is geometrically ergodic ([18]).

**• Likelihood Inference**

Suppose that *θ* denotes the three-dimensional vector of unknown parameters of Model (2) $\left[i.e.,\theta ={\left(d,a,b\right)}^{\prime }\right]$ and write the true value of the parameters as $\theta_{0}={\left(d_{0},a_{0},b_{0}\right)}^{\prime }$. We suppress the index *s* for the ease of presentation. Then, the conditional likelihood function for *θ*, given the starting value of $\lambda_{0}=exp\left(v_{o}\right)$ in terms of the observations $Y_{1},\ldots .Y_{n},$ is given by

$$L\left(\theta \right)=\overset{n}{\underset{t=1}{\prod }}\frac{exp(-\lambda_{t}\left(\theta \right))\lambda_{t}^{Y_{t}}(\theta )}{Y_{t}!}.$$

Hence, the log-likelihood function is given, up to a certain constant, by

$$l\left(\theta \right)=\overset{n}{\underset{t=1}{\sum }}l_{t}\left(\theta \right)=\overset{n}{\underset{t=1}{\sum }}(Y_{t}v_{t}\left(\theta \right)-exp\left(v_{t}(\theta \right))),$$

where $v_{t}\left(\theta \right)=d+av_{t-1}\left(\theta \right)+blog\left(1+Y_{t-1}\right)$. The score function is defined by

$$S_{n}\left(\theta \right)=\frac{\partial l\left(\theta \right)}{\partial \theta }=\overset{n}{\underset{t=1}{\sum }}\frac{\partial l_{t}\left(\theta \right)}{\partial \theta }=\overset{n}{\underset{t=1}{\sum }}\left(Y_{t}-exp\left(v_{t}\left(\theta \right)\right)\right)\frac{\partial v_{t}\left(\theta \right)}{\partial \theta },$$

where $\frac{\partial v_{t}}{\partial \theta }$ is a three-dimensional vector with components given by

$$\frac{\partial v_{t}}{\partial d}=1+a\frac{\partial v_{t-1}}{\partial d},\frac{\partial v_{t}}{\partial d}=v_{t-1}+a\frac{\partial v_{t-1}}{\partial a},\frac{\partial v_{t}}{\partial b}=log\left(1+Y_{t-1}\right)+a\frac{\partial v_{t-1}}{\partial b}.$$

The solution of the equation *S_n_*(*θ*) = 0, if it exists, yields the conditional MLE $\hat{\theta }$ of *θ*. Furthermore, the Hessian matrix for Model (2) is obtained by further differentiation of the following score equation:

$$H_{n}\left(\theta \right)=-\overset{n}{\underset{t=1}{\sum }}\frac{\partial^{2}l_{t}\left(\theta \right)}{\partial \theta \partial \theta^{\prime }}$$

$$\overset{n}{\underset{t=1}{\sum }}\frac{Y_{t}}{\lambda_{t}^{2}\left(\theta \right)}\left(\frac{\partial \lambda_{t}\left(\theta \right)}{\left(\theta \right)}\right){\left(\frac{\partial \lambda_{t}\left(\theta \right)}{\partial \left(\theta \right)}\right)}^{\prime }-\overset{n}{\underset{t=1}{\sum }}\left(\frac{Y_{t}}{\lambda_{t}\left(\theta \right)}-1\right)\left(\frac{\partial^{2}\lambda_{t}\left(\theta \right)}{\partial \theta \partial \theta^{\prime }}\right).$$

The model (2) can be estimated by a maximum likelihood method ([18]).

## 4. Results

**Figure 1.**
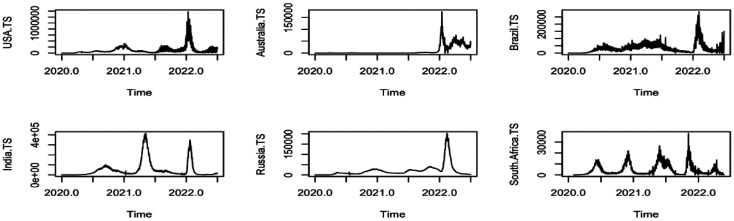
Areal trajectories of confirmed cases per day.

**Figure 2.**
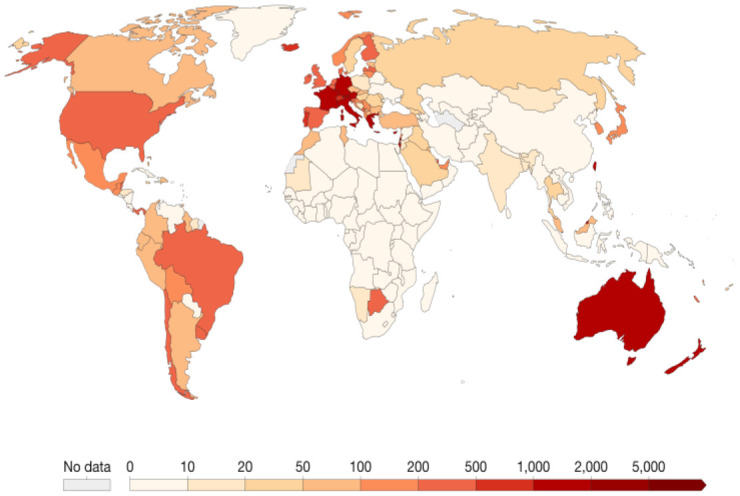
Geographical mapping of confirmed cases globally as of June 2022.

We worked with the data spanning the day the virus was scientifically recorded in Wuhan, China until the end of June 30, 2022. Figure 1 shows the areal unit-based trajectories, and Figure 2 shows the geographical distribution of the virus by the end of June 30, 2022, with associated Autocorrelation Functions (ACFs) (Figure 1 in Appendix). The Pearson correlation between confirmed cases and tests performed was found to be significant (correlation = 0.9062, *P − value* = 0.034).

**Figure 3.**
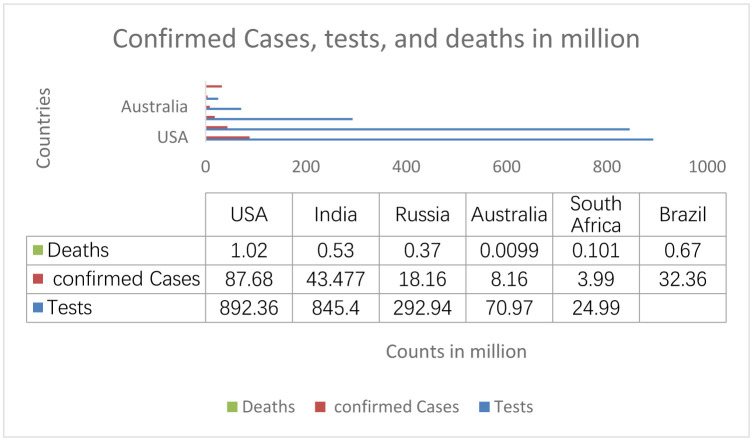
Tests, confirmed cases and deaths.

From Figure 3, the USA has the highest number of COVID-19 cases, followed by India, Brazil, Russia, Australia and South Africa, respectively. The country with the highest number of tests per million people was again the USA. India was the second country with the highest number of tests, followed by Russia and South Africa, respectively. Data were not available for Brazil for that date.

**Figure 4.**
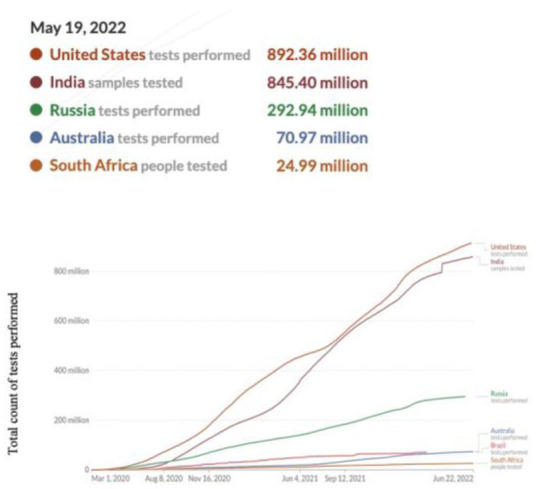
Cumulative COVID-19 tests performed.

Figure 4 shows the trend of tests performed. The graph indicates an increasing trend of tests performed as time passed. By May 19, 2022, the USA had the highest number of tests performed and South Africa had the lowest number of tests performed.

**Figure 5.**
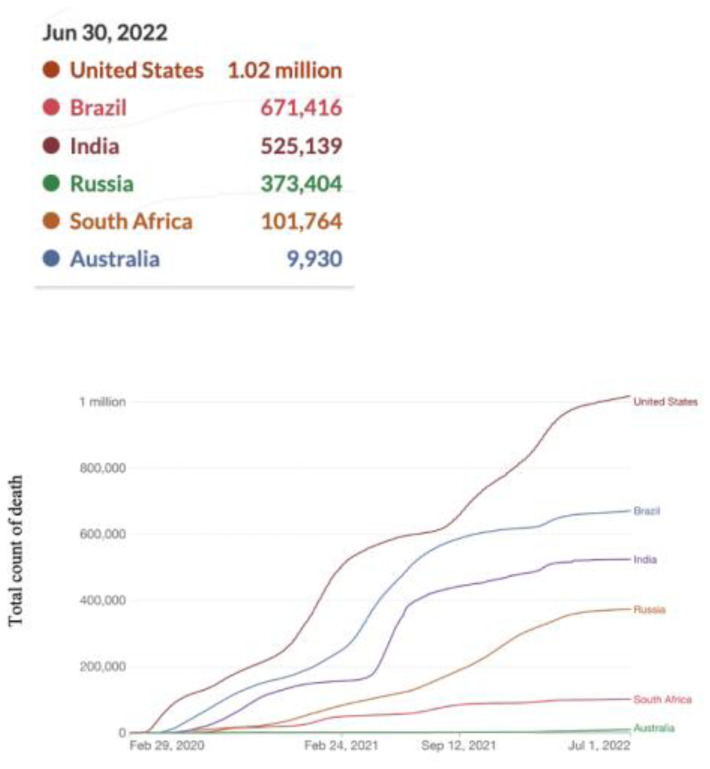
Cumulative COVID-19 deaths.

Figure 5 shows the trend of deaths recorded as of June 30, 2022. The USA had the highest recorded number of deaths, Brazil had 671,416 deaths recorded, taking the second position, and Australia had the fewest deaths.

**Figure 6.**
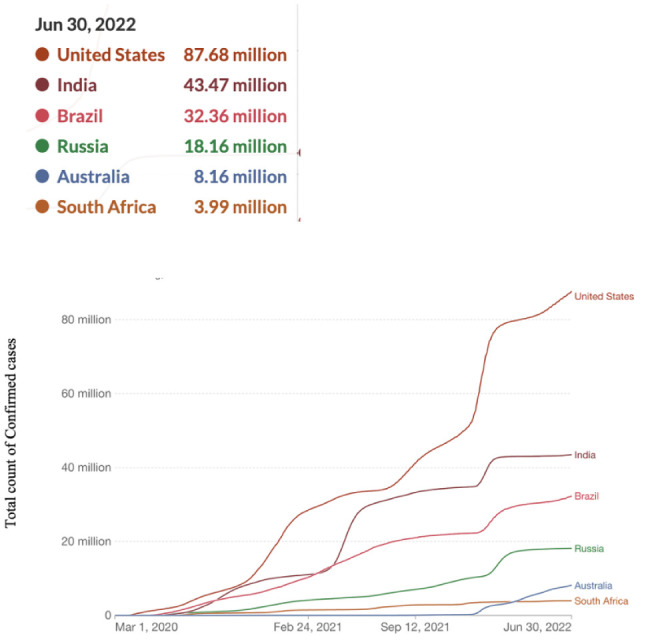
Cumulative COVID-19 confirmed cases.

Figure 6 shows the total confirmed cases recorded until June 30, 2022. The graph indicates that the USA had the highest number of confirmed cases recorded, followed by India, Brazil, Russia, Australia, and South Africa, respectively.

**Figure 7.**
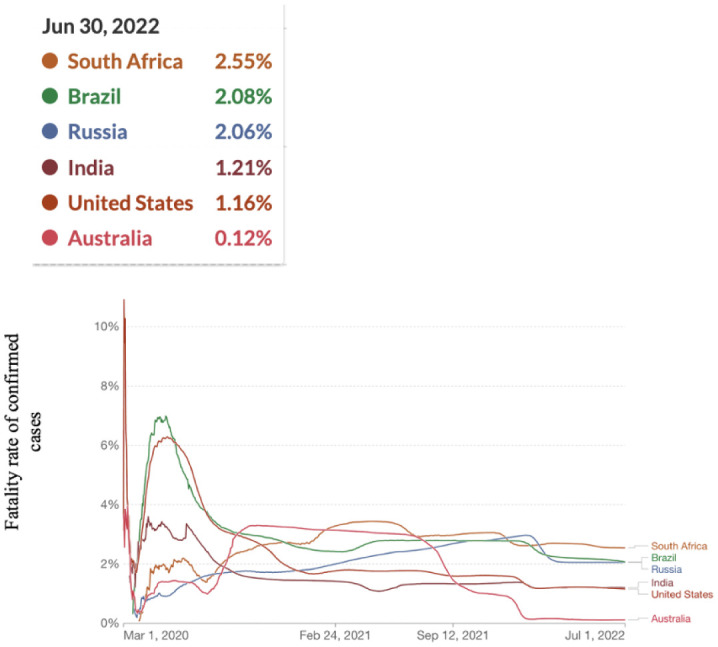
Fatality rate for COVID-19.

Figure 7 shows that South Africa had the highest fatality rate. Brazil had the second highest fatality rate, followed by Russia, India, the USA and Australia, which had the lowest fatality rate. The trend of fatality indicates a decreasing trend. The fatality rate was high between March 2020 and June 2020, but it started to decline from February 2021.

Table 2 below shows the estimated autoregressive coefficients for the log-linear PAR models and its associated 95% confidence intervals. The *α* component in the model indicates the short-term dependence on the observed cases from the previous day, and the *β* component represents the long-term trend component, i.e., the long-term trend. The estimated coefficients until June 30, 2022 indicate the presence of both short-term dependence on the previous cases and a feedback mechanism for the countries under study; however, only the presence of a long-term trend and no substantial short-term dependence was observed for Russia; *α* > *β* (Table 2) in the USA, South Africa and Australia, and *β* > *α* (Table 2) in India, Brazil and Russia.

**Table 2．:** Areal unit-based parameter estimates of confirmed cases.

		**Estimate**	**Std. error**	**CI (lower)**	**CI (upper)**	**AIC**
**USA**	**(Intercept)**	0.253	0.000406	0.252	0.254	18290779.0
	*β*	0.264	0.000159	0.264	0.265	
	*α*	0.715	0.000171	0.715	0.716	
**INDIA**	**(Intercept)**	0.706	2.20*10^−3^	7.02*10^−1^	7.10*10^−1^	1527702.0
	*β*	0.571	8.20*10^−4^	5.70*10^−1^	5.73*10^−1^	
	*α*	0.360	8.49*10^−4^	3.58*10^−10^	3.62*10^−1^	
**SOUTH AFRICA**	**(Intercept)**	2.680	2.20*10^−3^	7.02*10^−1^	7.10*10^−1^	842091.2
	*β*	0.213	8.20*10^−4^	5.70*10^−1^	5.73*10^−1^	
	*α*	0.435	8.49*10^−4^	3.58*10^−10^	3.62*10^−1^	
**BRAZIL**	**(Intercept)**	9.650	2.20*10^−3^	7.02*10^−1^	7.10*10^−1^	12592541.0
	*β*	0.250	8.20*10^−4^	5.70*10^−1^	5.73*10^−1^	
	*α*	−0.222	8.49*10^−4^	3.58*10^−10^	3.62*10^−1^	
**AUSTRALIA**	**(Intercept)**	0.657	2.20*10^−3^	7.02*10^−1^	7.10*10^−1^	1639427.0
	*β*	0.249	8.20*10^−4^	5.70*10^−1^	5.73*10^−1^	
	*α*	0.682	8.49*10^−4^	3.58*10^−10^	3.62*10^−1^	
**RUSSIA**	**(Intercept)**	0.376	2.20*10^−3^	7.02*10^−1^	7.10*10^−1^	70869.0
	*β*	0.895	8.20*10^−4^	5.70*10^−1^	5.73*10^−1^	
	*α*	0.065	8.49*10^−4^	3.58*10^−10^	3.62*10^−1^	

## 5. Discussion

In the USA, if the expected number of new cases for yesterday was close to zero, 100 new cases observed the day before would generate about 26 new expected cases today. If no cases were observed yesterday, an expectation of 100 new cases for yesterday generates approximately 3 new expected cases today, based on the value estimated for *β*. Furthermore, we note that the AIC was relatively high for the goodness-of-fit; particularly, it was the highest among all of the countries. In general, we anticipated a lower AIC for the model.

In the case of India, if the expectation of new cases for the previous day was close to zero, 100 new cases observed the day before would generate about 5 new expected cases today. If no cases were observed yesterday, an expectation of 100 new cases for the previous day generates approximately 14 new expected cases today.

In South Africa, if the previous day's expected number of new cases was close to zero, 100 new cases observed the previous day would generate about 7 new expected cases today. If no cases were observed yesterday, an expectation of 100 new cases for the previous day generates approximately 3 new expected cases today. South Africa had one of the lowest AICs of any country.

For Brazil, if the previous day's expected number of new cases was close to zero, 100 new cases observed the previous day would generate about 1 new expected case today. If no cases were observed yesterday, an expectation of 100 new cases for the previous day generates approximately 3 new expected cases today.

In the case of Australia, if the previous day's expected number of new cases was close to zero, 100 new cases observed the previous day would generate about 23 new expected cases today. If no cases were observed yesterday, an expectation of 100 new cases for the previous day generates approximately 3 new expected cases today.

Finally, for Russia, if the previous day's expected number of new cases was close to zero, 100 new cases observed the previous day would generate about 2 new expected cases today. If no cases were observed yesterday, an expectation of 100 new cases for the previous day generates approximately 62 new expected cases today. Russia's AIC appears to be the best because it is the lowest of the countries.

According to the research, if the expected number of new cases for the previous day was close to zero, Russia will have the most cases today, with Australia, South Africa and Brazil having the fewest. The study also found that the fatality rate was high in South Africa and Brazil. Australia had the lowest fatality rate among the six countries. The COVID-19 strains rapidly evolved from Alpha to Delta, then to Omicron; this could be attributed to different country-based public health non-pharmaceutical policies. As compared to other pandemics, the COVID-19 pandemic has one of the lowest fatality rates (3.4%). Severe acute respiratory syndrome (SARS) has been associated with a fatality rate of 15%, and Ebola has been associated with a fatality rate of 50%.

This study contributes to the literature by helping us to ascertain a predictive model of the dynamics of the outbreak of coronavirus from January 2020 to June 2022. This model-based study can aid in predicting the impact of a given intervention on the spread of an outbreak when the preventive measures with the data of percentage of vaccinated population taken by each country under study are further examined. According to existing research, public relations practitioners and researchers use non-pharmaceutical measures such as geographic areas, social economic factors such as income, ethnicity and mobility, and public relations strategies and campaigns to reduce COVID-19 cases around the world. It also helps policymakers implement measures to provide a conducive environment and support for human resources, vaccination against the virus's spread. Recent research has presented the use of a particular state-of-the-art quantitative method with emphasis on the latent log-linear Poisson processes modeling of exponential growth for observational studies, which allows for the examination of multiple areal units and consideration of multivariate interaction. This will drive the policymaking concerning mitigating the spread of the virus. Institutions and organizations will benefit significantly since the findings from the model will aid employers and management in instituting measures that will help provide a conducive environment and support for all workers/staff/students. It will provide guidance to prospective workers in the fields of nursing, teaching, hospitality and transport about the nature of their future jobs and what to expect concerning the spread of the virus.

## 6. Conclusion

This study exploited a class of log-linear Poisson models for modeling the co-occurrences of counting processes by using a structure that can parameterize both positive and negative associations. The inclusion of the *β* component in counting processes allows us to capture long-term memory effects, and it is analogous to moving from an ARCH to a GARCH model using Gaussian processes.

The current study demonstrated a spatial statistical approach capable of surveying multiple areal units (at this time, focusing on data at the country level) and supporting multivariate interaction. It can be used to incorporate other public health-related parameters for further analysis at a finer-grained areal level, such as city or county. Other socioeconomic variables, such as ethnicity, gender, health and mobility [10],[22], can be examined alongside this statistical model. To understand pandemic spreading and disease control, environmental and geographical factors such as weather, temperatures, sewage and watercourses can be considered together. New data sources derived from wastewater location-based COVID-19 detection, as well as public health data based on geographic units, can be combined to analyze virus spread patterns and provide recommendations for health, crisis management and social policies, as well as how nations can prevent future pandemics through vaccination and non-pharmaceutical interventions in the context of good governance.

Although the temporal dependence was taken into account, the analysis relies on the assumption of spatial independence on the areal units based on the selection of each areal unit from distinct continents to mitigate the spatial dependence. In terms of academia, this research study serves as our starting point for spatiotemporal dependence in the coming research on the complexity and change points of spatiotemporal dynamics with respect to continuously indexed space and time through the use of high-dimensional statistical methods. The recommendations from the study for future research can benefit researchers who wish to delve more into modeling the dynamics of the spread of the virus with higher predictive accuracy.
