# Supplementary material for: Temporal dynamics for areal unit-based co-occurrence COVID-19 trajectories
Source: AIMS Public Health. 2022 Oct 14;9(4):703–17. doi: 10.3934/publichealth.2022049 (PMC9807409; doi:10.3934/publichealth.2022049)
Supplement: Supplementary file 1 [file publichealth-09-04-049-s001.pdf]

AIMS Public Health

http://www.aimspress.com/journal/aimsph

AIMS Public Health, 9(4): 703–717.
DOI:10.3934/publichealth.2022049
Received: 20 April 2022
Revised: 17 August 2022
Accepted: 01 September 2022
Published: 14 October 2022

Research article

Temporal dynamics for areal unit-based co-occurrence COVID-19 trajectories

Gabriel Owusu[1], Han Yu[1*] and Hong Huang[2]

[1] Department of Applied Statistics and Research Methods, University of Northern Colorado, Greeley, CO 80639, USA
[2] School of Information, University of South Florida, Tampa, FL, 33620, USA

* **Correspondence:** Email: han.yu@unco.edu.

Proposition 1 (Fokianos and Tjøstheim, 2011**)**. Assume

$$Y_t^m = N_t(\lambda_t^m) = N_t\left(exp(v_t^m)\right), v_t^m = d + av_{t-1}^m + blog(Y_{t-1}^m + 1) + \epsilon_{t,m},$$

with v_0^m, Y_0^m fixed, where $\{N_t(.)\}$ is identical to the sequence $\{N_t(.)\}$ of $Y_t = N_t(\lambda_t), v_t = d + av_{t-1} + blog(Y_{t-1} + 1)$ and $\epsilon_{t,m} = c_m 1(Y_{t-1}^m = 1)U_t, c_m > 0, c_m \longrightarrow 0\ as\ m \longrightarrow \infty$.
Suppose that $|a| < 1$. In addition, assume that $b > 0$ for $|a + b| < 1$, and that $b < 0$ for $|a||a + b| < 1$. Then, the following conclusions hold:

1. The process $\{v_t^m, t \geq 0\}$ is a geometrically ergodic Markov chain with finite moments of order k for an arbitrary k.
2. The process $\{(Y_t^m, U_t, v_t^m), t \geq 0\}$ is a $V_{(Y,U,v)}$-geometrically ergodic chain with $V_{Y,U,\lambda}(Y, U, v) = 1 + log^{2k}(1 + Y) + v^{2k} + U^{2k}, k$ being a positive integer.

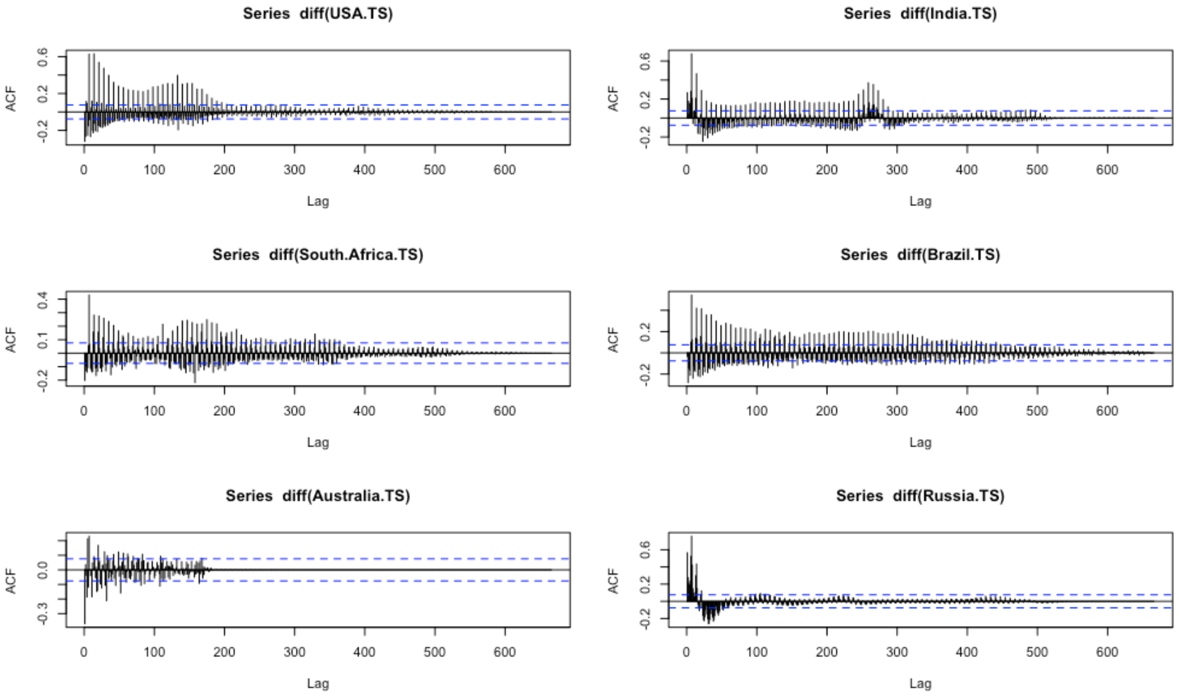

Figure 1: ACFs of areal trajectories
